# Association of socio‐demographic factors and personal hygiene with infectious childhood dermatoses

**DOI:** 10.1002/ski2.219

**Published:** 2023-02-10

**Authors:** Raksha Pathak, Sameer Shrestha, Prakash Poudel, Suchana Marahatta, Dhan Keshar Khadka

**Affiliations:** ^1^ Department of Dermatology and Venereology B.P Koirala Institute of Health Sciences Dharan Nepal; ^2^ Department of Pediatrics and Adolescent Health B.P Koirala Institute of Health Sciences Dharan Nepal

## Abstract

**Background:**

Paediatric dermatoses vary vastly from adult dermatoses in terms of clinical presentation, management, and prognosis thereby generating special interest in this field. Many factors like geographical area, climatic exposure, seasons, culture, socioeconomic factors, hygiene, dietary habits, literacy influences pattern of skin diseases in children resulting in marked variation in prevalence and pattern.

**Objectives:**

This study aims to find out association of socio‐demographic factors and personal hygiene with infectious childhood dermatoses.

**Materials and Methods:**

This was a hospital based cross‐sectional study. All children (0–14 years) attending Dermatology outpatient clinics were enroled. Proforma was prepared to assess the socio‐demographic factors and personal hygiene in the local context. Chi‐square test was applied to find the association of the baseline variables with infectious dermatoses.

**Results:**

A total of 364 children were enroled with mean age of 6.97 ± 4.23 years. Females (52.5%) were slightly more than males (47.5%). There were 201 (55.2%) infectious and 163 (44.8%) non‐infectious dermatoses. Bacterial infections were the most common group (18.7%), followed by dermatitis and eczemas (14.0%) and viral infections (13.7%). Lower educational status of mother (*p* = 0.025), lower monthly family income (*p* = 0.008), lower socioeconomic status (*p* = 0.015) and less frequent bathing habits (*p* = 0.014) were associated with increased risk of infective dermatoses.

**Conclusion:**

Infections and infestations were the most common paediatric dermatoses in our Outpatient Department. Female education, upliftment of socioeconomic status of family and improving personal hygiene may reduce the risk of skin infections in children.

## Graphical abstract

Infections and infestations were the most common paediatric dermatoses in our Outpatient Department. Lower educational status of mother (*p* = 0.025), lower monthly family income (*p* = 0.008), lower socioeconomic status (*p* = 0.015) and less frequent bathing habits (*p* = 0.014) were associated with increased risk of infective dermatoses. Female education, upliftment of socioeconomic status of family and improving personal hygiene may reduce the risk of skin infections in children.

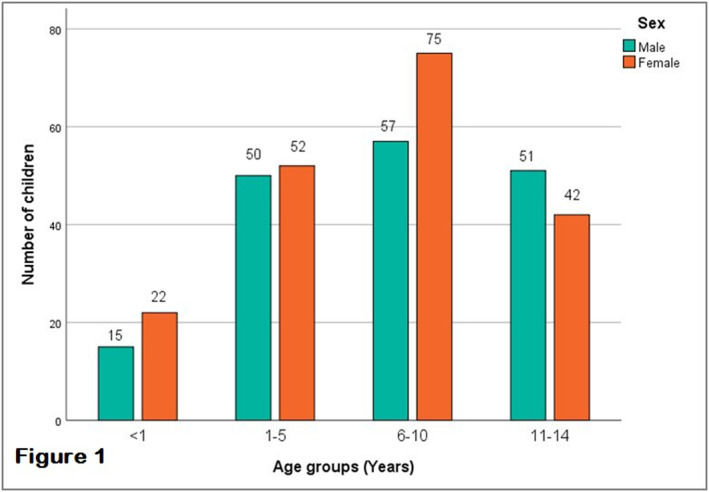

1

**What is already known about this topic?**

- Infections and infestations are the most common dermatoses in children in developing countries.

**What does this study add?**

- Lower educational status of mother, low family income, lower socioeconomic status and less frequent bathing habits increase the risk of infections in children.

## 2 INTRODUCTION

Paediatric dermatology is a branch of dermatology that deals with diagnosis, treatment, and prevention of skin diseases in infancy, childhood and adolescence.^1^ Paediatric dermatoses vary from adult dermatoses in terms of clinical presentation, management and prognosis and they carry significant burden on quality of life.^2^ The dermatological problems constitute about 30% of all outpatient visits to pediatricians and 30% of all dermatological outdoor consultation involve children.^3^ Skin of children is more susceptible to infections and infestations due to immature immune system and more exposure to sub‐clinical infectious carriers in school and within the family.^4^

The prevalence and pattern of skin diseases in paediatric age group differ from one country to another and within the same country from one region to another.^5^ Many factors like geographical area, climatic exposure, seasons, culture, socioeconomic factors, hygiene, dietary habits, illiteracy influences prevalence and pattern of skin diseases in children.^6^ Studies from the Middle East and Western countries have shown dermatitis as the most prevalent paediatric dermatoses^1^, ^6^, ^7^, ^8^, ^9^ while infections and infestations are most common in developing countries.^5^, ^10^, ^11^, ^12^, ^13^, ^14^, ^15^

Many studies on paediatric dermatoses have been conducted in other parts of the world and in other parts of Nepal. The pattern of dermatoses and the associated risk factors are important for management plan and prevention strategies. However, no published work is available from Eastern Nepal. Hence we aimed to identify the pattern of dermatoses in children attending Dermatology Outpatient Department clinic and to investigate the association of baseline variables with infective diseases.

## 3 MATERIALS AND METHODS

In this hospital based cross‐sectional study, 364 children (0–14 years) attending dermatology OPD of B.P. Koirala Institute of Health Sciences were enroled from March 2020 to February 2021 with consecutive sampling method. The purpose of study was explained to the parents before enrolment in the study. The participant information sheet and informed consent form were given to parents to read or were read by the doctors in Nepali language, if he/she was illiterate. The information was collected and written by the doctors themselves in predesigned performa. Ethical clearance was taken from Institutional Review Committee.

The patients were divided into two groups—Patients with ‘infectious dermatoses’ and ‘non‐infectious dermatoses’. Infectious dermatoses are defined as those skin disorders that are caused by microorganisms such as bacteria, viruses, parasites or fungi and can spread directly or indirectly from one individual to another. The diagnosis was made with history, clinical examinations and required investigations. Patients who had both infectious and non‐infectious dermatoses were excluded in the study. Age was categorized as neonates (0–28 days), infants (<1 year), pre‐school children (1–5 years), school children (6–10 years) and adolescents (11–14 years). Socio‐demographic data including age, sex, educational status and occupation of parents, gross monthly income, family size, birth order, socioeconomic status (using Kuppuswamy's scale modified in context of Nepal^16^) and hygiene practices including frequency of bathing, frequency of washing hands, towel sharing, sharing of clothes, frequency of nail trimming were recorded and compared among the groups.

Data were entered into MS Excel 2010 and converted to Statistical Package for Social Sciences (SPSS) version 11.5 for statistical analysis. Continuous variables were expressed as mean (±SD). Categorical variables were expressed in percentage or proportion. Mean (±SD), percentage, proportion, frequency were used for descriptive statistics. For inferential analysis, Chi square test was used where *p* value of <0.05 was considered statistically significant.

## 4 RESULTS

A total of 13,107 patients visited our OPD during the study period. Among them, 2149 (16.4%) were paediatric cases. However, only 364 children were enroled in our study.

**FIGURE 1:**
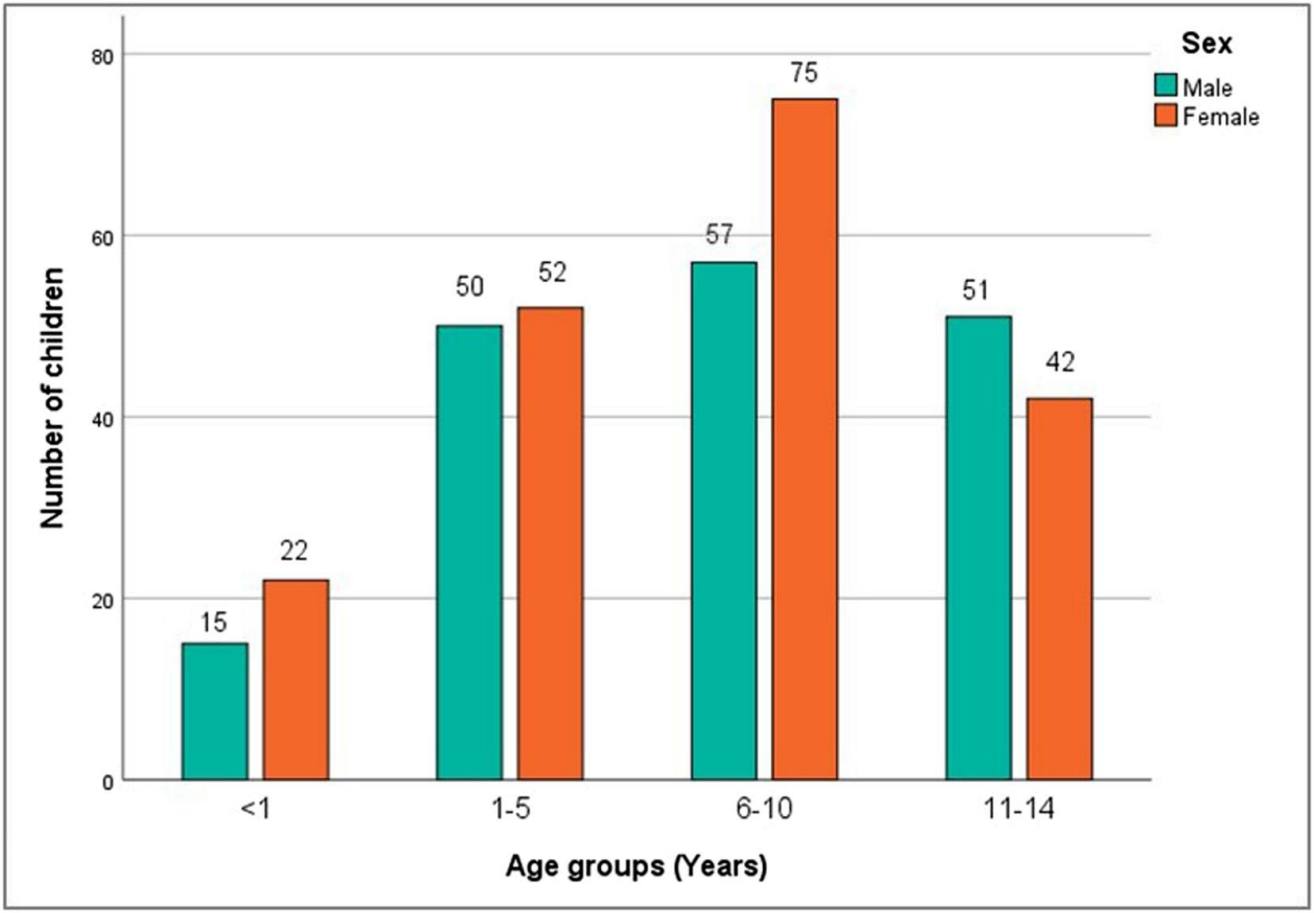
Age and gender distribution of participants.

The age of the patients ranged from 1 day to 14 years with mean age 6.97 ± 4.23 years. Majority of the children (36.3%) were school children followed by preschool children (28.0%), adolescents (25.5%) and infants (10.2%). Females were more than males with ratio of 1: 1.1 (Figure 1).

### 4.1 Disease pattern

Infectious dermatoses (*n* = 201, 55.2%) predominated the non‐infectious dermatoses (*n* = 163, 44.8%). Bacterial infections were most prevalent (18.7%), followed by dermatitis and eczema (14.0%), viral infections (13.7%), fungal infections (11.5%) and infestations (11.3%). Five most common diseases were scabies (10.4%), impetigo contagiosa (7.4%), atopic dermatitis (6.3%), urticaria (6.3%) and common wart (5.8%) (Tables 1 and 2).

**TABLE 1:** Patten of infectious dermatoses.

Category	Sub‐category	Frequency *n* (%)
Bacterial	Impetigo contagiosa	27 (7.4)
	Furuncle	15 (4.1)
	Folliculitis	8 (2.2)
	Bullous impetigo	8 (2.2)
	Cellulitis	3 (0.8)
	Abscess	3 (0.8)
	Secondary syphilis	2 (0.5)
	Leprosy	1 (0.3)
	Streptococcal vulvovaginitis	1 (0.3)
	Total	68 (18.7)
Viral	Wart	21 (5.8)
	Molluscum contagiosum	14 (3.8)
	Varicella	7 (1.9)
	Hand foot mouth disease	2 (0.5)
	Herpes labialis	1 (0.3)
	Viral exanthem (others)	5 (1.4)
	Total	50 (13.7)
Fungal	Tinea corporis	17 (4.7)
	Pityriasis versicolour	7 (1.9)
	Tinea cruris	6 (1.6)
	Tinea capitis	5 (1.4)
	Candidiasis	4 (1.1)
	Tinea facei	3 (0.8)
	Total	42 (11.5)
Parasitic	Scabies	38 (10.4)
	Pediculosis	3 (0.8)
	Total	41 (11.3)

**TABLE 2:** Pattern of non‐infectious dermatoses.

Category	Sub‐category	Frequency *n* (%)
Dermatitis and eczema	Atopic dermatitis	23 (6.3)
	Seborreic dermatitis	8 (2.2)
	Pityriasis alba	7 (1.9)
	Nummular dermatitis	6 (1.6)
	Contact dermatitis	5 (1.4)
	Pompholyx	2 (0.5)
	Total	51 (14.0)
Urticaria	Urticaria	23 (6.3)
Stings and bites	IBH/papular urticaria	13 (3.6)
Sweat glands disorders	Miliaria rubra	12 (3.3)
Genetic disorders	Epidermal nevus	3 (0.8)
	Melanocytic nevus	3 (0.8)
	Neurofibromatosis 2	2 (0.5)
	Aplasia cutis congenita	1 (0.3)
	Epidermolysis bullosa simplex	1 (0.3)
	Total	10 (2.7)
Sebaceous glands disorders	Acne vulgaris	6 (1.6)
	Sebaceous hyperplasia	1 (0.3)
	Total	7 (1.9)
Papulosquamous disorders	Psoriasis	2 (0.5)
	Lichen planus	2 (0.5)
	Lichen nitidus	1 (0.3)
	Lichen striatus	1 (0.3)
	Pityriasis rubra pilaris	1 (0.3)
	Total	7 (1.9)
Collagen vascular disease	Systemic lupus erythematosus	2 (0.5)
	Morphea	2 (0.5)
	Henoch schonlein purpura	2 (0.5)
	Acute haemorrhagic oedema of infancy	1 (0.3)
	Total	7 (1.9)
Cyst/tumour	Infantile haemangioma	3 (0.8)
	Mucosal cyst	2 (0.5)
	Milia	2 (0.5)
	Total	7 (1.9)
Adverse drug reaction	Drug induced exanthem	6 (1.6)
Pigmentary disorders	Vitiligo	4 (1.1)
	Mongolian spot	1 (0.3)
	Total	5 (1.4)
Hair disorders	Alopecia areata	4 (1.1)
Nutritional disorders	Phrynoderma	2 (0.5)
	Acrodermatitis enteropathica	2 (0.5)
	Total	4 (1.1)
Nail disorders	Onychophagia	2 (0.5)
Bullous disorders	Chronic bullous disease of childhood	1 (0.3)
Photodermatoses	Polymorphous light eruption	1 (0.3)
Others	Urticaria pigmentosa	2 (0.5)
	Erythema toxicum neonatorum	1 (0.3)
	Total	3 (0.8)

### 4.2 Association of socio‐demographic characteristics with infectious dermatoses

Non‐infectious dermatoses were common in infants whereas infectious dermatoses were common in age groups above 1 year (*p* = 0.027). Bacterial infections were common among school children (*p* = 0.321). Viral infections and parasitic infestations were prevalent among adolescents (*p* = 0.107) and preschool children (*p* = 0.195) respectively. Infections were more common in children whose mothers have lower educational status (*p* = 0.025) but education and occupation of father and occupation of mother had no relation with prevalence of infectious diseases. Lower monthly family income (*p* = 0.008) and lower socioeconomic status (*p* = 0.015) were significantly associated with increased risk of infectious diseases but birth order of child and family size did not affect occurrence of infectious diseases (Table 3).

**TABLE 3:** Comparision of infectious and non‐infectious dermatoses with sociodemographic characteristics.

Charateristics	Category	Infections and infestations *n* (%)	Non‐infectious *n* (%)	ϰ^2^	*p* Value
Age group	<1 year	12 (5.9)	25 (15.3)	9.203	0.027
	1–5 years	62 (30.8)	40 (24.5)		
	6–10 years	74 (36.8)	58 (35.6)		
	11–14 years	53 (26.4)	40 (24.5)		
Sex	Male	98 (48.8)	75 (46.0)	0.272	0.602
	Female	103 (51.2)	88 (54.0)		
Father's education	Primary level	84 (41.8)	67 (41.1)	0.017	0.895
	Secondary level and above	117 (58.2)	96 (58.9)		
Mother's education	Primary level	100 (49.8)	62 (38.0)	5.001	0.025
	Secondary level and above	101 (50.2)	101 (62.0)		
Father's occupation	Professionals	47 (23.4)	42 (25.8)	3.838	0.699
	Business	25 (12.4)	19 (11.7)		
	Skilled worker	36 (17.9)	27 (16.6)		
	Unskilled worker	25 (12.4)	16 (9.8)		
	Farmer	48 (23.9)	49 (30.1)		
	Unemployed	11 (5.5)	5 (3.1)		
	Student	9 (4.5)	5 (3.1)		
Mother's occupation	Professionals	16 (8.0)	20 (12.3)	5.069	0.535
	Business	16 (8.0)	16 (9.8)		
	Skilled worker	32 (15.9)	31 (19.0)		
	Unskilled worker	17 (8.5)	11 (6.7)		
	Farmer	51 (25.4)	32 (19.6)		
	Housewife	62 (30.8)	50 (30.7)		
	Student	7 (3.5)	3 (1.8)		
Birth order	1st	76 (37.8)	52 (31.9)	3.908	0.421
	2nd	96 (47.8)	75 (46.0)		
	Subsequent	29 (14.4)	36 (22.1)		
Family size	≤5	140 (69.7)	107 (65.6)	0.663	0.416
	>5	61 (30.3)	56 (34.4)		
Monthly family income	<30 000	97 (48.3)	56 (34.4)	7.140	0.008
	≥30 000	104 (51.7)	107 (65.6)		
Socioeconomic status	Upper	11 (5.5)	16 (9.8)	12.363	0.015
	Upper middle	37 (18.4)	47 (28.8)		
	Lower middle	78 (38.8)	61 (37.4)		
	Upper lower	40 (19.9)	24 (14.7)		
	Lower	35 (17.4)	15 (9.2)		

### 4.3 Association of personal hygiene with infectious dermatoses

Risk of infectious dermatoses increases with less frequent bathing (*p* = 0.014). Whereas, it was not affected by the frequency of hand washing, nail trimming, and towels or clothes sharing (Table 4).

**TABLE 4:** Comparision of infectious and non‐infectious dermatoses with personal hygiene.

Charateristics	Category	Infections and infestations *n* (%)	Non‐infectious *n* (%)	ϰ^2^	*p* Value
Frequency of bathing	Daily	31 (15.4)	36 (22.1)	8.512	0.014
	>Once weekly	63 (31.3)	65 (39.9)		
	Once weekly or less frequently	107 (53.2)	62 (38.0)		
Frequency of washing hands	≤5	63 (31.3)	56 (34.4)	0.844	0.656
	6–10	91 (45.3)	75 (46.0)		
	>10	47 (23.4)	32 (19.6)		
Nail trimming	≤2 weeks	91 (45.3)	81 (49.7)	0.705	0.401
	Less frequently	110 (54.7)	82 (50.3)		
Sharing towels	Yes	163 (81.1)	121 (74.2)	2.471	0.116
Sharing clothes	Yes	118 (58.7)	100 (61.3)	0.262	0.609

## 5 DISCUSSION

Age, sex, birth order, family size, overcrowding, type of housing, rural residence, lack of parental education, lower socioeconomic status, poor nutrition and personal hygiene have been linked to increased incidence of skin infections in children. This is one of the few studies which have tried to access these associations.

Though paediatric dermatoses put a large burden on health care system, little is known about prevalence of paediatric dermatoses in community as most of the studies are hospital based. In different parts of the world, prevalence of skin diseases in children varies from 4.6% to 87%.^6^, ^10^, ^11^, ^12^, ^13^ In Nepal, few hospital based studies are conducted showing prevalence of 12%–22.64% comparable to the finding in our study (16.4%).^2^, ^17^, ^18^, ^19^, ^20^ Infections and infestations were most common dermatoses similar to other studies from Nepal and other developing countries.^2^, ^5^, ^10^, ^11^, ^12^, ^13^, ^14^, ^15^, ^17^, ^18^, ^21^, ^22^, ^23^

### 5.1 Association with socio‐demographic factors

Age has significant association with occurrence of various diseases. In infants, number of non‐infectious dermatoses was higher than infectious dermatoses and infectious dermatoses were common in older children and adolescents similar to other studies.^1^, ^18^, ^22^, ^23^, ^24^ This might be because of extensive care of infants at home and less exposure in the community whereas older children have increased exposure of possible contacts due to overcrowding in schools and increase in outdoor and sports activities.

The study does not show association of sex with increased risk of infestions similar to other studies.^25^, ^26^ This could be a positive sign that even girls are given equal care as boys these days. Neupane S et al., Poudyal Y et al. and Amoran OE et al. have accounted higher prevalence of infections in males^17^, ^18^, ^27^ in contrast to Bilal MD et al. and Libu GK et al., who reported that girls had significantly higher prevalence than boys.^6^, ^28^

The high prevalence of infections may be an indicator of child neglect with poor parental supervision, negligence in hygiene and nutrition. It is expected that with increased birth other and increased family size, the care of child is compromised. Birth order correlated positively with risk of infections and infestations in some studies^27^, ^29^ but others have shown no significant association^21^, ^28^ in concordance to our study. Family size is an important factor that is related to birth order, family type, household density, housing status and socioeconomic status in one way or the other. Transmissible skin diseases are observed more frequently in families with larger family size and overcrowding.^21^, ^28^, ^30^ However, the prevalence of infectious skin disorders did not yield significant association with family size in our study. Even with larger family size, if good housing provisions, hygiene practices, nutrition are maintained, risk of acquiring infectious diseases might be less.

Studies have shown variable results on relation of parental education with prevalence of infectious diseases. Increased prevalence of skin infections and infestations associated with lower parental education was seen in various studies^21^, ^28^, ^29^, ^31^ but others have shown no association.^25^, ^27^, ^32^ In our study we have found that educational status of mother has inverse relation to prevalence of infections and infestations but fathers' education has no association. Since child's hygiene, nutrition, supervision are often taken care by mothers and child's health is directly related to mother's health, the finding was not surprising. Educated mothers are better able to prevent diseases and promote the health of their children. So, female education can play role in control of infectious diseases in children.

Oyedeji OA have shown increased prevalence of infectious disease associated with lower parental occupational status among primary school children.^31^ However, Amoran et al. shows no relation.^27^ A hospital based study in Nepal have shown no association of fathers occupation with prevalence of infections in children similar to our study but increased risk of infection in children whose mothers were housewives in contrary to our result.^21^ Though, occupational status is directly related to parental education, income and socioeconomic status, other factors might be more important and reduced the significance of its influence on prevalence of infectious diseases. Variation in categories of occupations in different studies might have shown the variable results. This may need further investigation.

Infectious diseases were common in families with lower monthly income showing significant association similar to Inanir I et al.^29^ and contrary to other studies.^21^, ^25^ Risk of infections and infestations increases with lower socioeconomic status. Kharel C et al. reported majority of the children with pyoderma belonged to lower class family.^33^ Gauchan E et al. and Podder I et al. have also shown similar findings^21^, ^31^, ^34^ but some other studies have suggested no significant association.^26^, ^28^, ^32^

As, occupational and educational status, family income and socioeconomic status are inter‐related, it is not surprising that the results obtained for educational status, family income and socioeconomic status are similar.

### 5.2 Association with personal hygiene

Studies have suggested that children with poor personal hygiene are more vulnerable to develop skin infections.^25^, ^26^, ^27^, ^30^, ^32^ In most of the studies, the personal hygiene practices that were taken into consideration were sharing of items, bathing, hand‐washing, washing clothes, hair combing, trimming nails. However, the individual practices were not assessed separately. Amoran OE reported positive relation with towel sharing habits and risk of infections.^27^ Gauchan E et al. failed to prove the association of frequency of bathing, nail trimming and sharing of towels with infectious skin diseases.^21^

In this study, frequency of bathing, hand washing, nail trimming and habit of sharing towels and clothes were taken into account for the assessment of personal hygiene. Risk of infections and infestations in children increases significantly with less frequent bathing but not with lower frequency of hand‐washing and nail‐trimming. Hand hygiene is a key component of good hygiene practice that can contribute in reduction of transmission of infections.^35^ However, since we have not considered hand hygiene practices of the parents, hygiene practices of care‐takers specially the mothers might be more important than that of children. There was no significant difference in prevalence of infectious and non‐infectious diseases due to habit of sharing towels and clothes among family and friends. Washing of clothes and towels and drying in sunlight could probably decrease the risk of transmission of infections. So, improving personal hygiene practices of children as well as the family members can break the chain of transmission and reduce the risk of infection in children.

## 6 CONCLUSION

Infections and infestations were the most common paediatric dermatoses among those visiting dermatology OPD. School going age group, lower educational status of mother, low family income, lower socioeconomic status and less frequent bathing habits were found to be associated with increased frequency of infections and infestations. The public health strategies intended to control skin infections among children should include school health programs, female education and empowerment, uplifting socioeconomic status of families, safer sanitation and hygiene and quality health care facilities.

### 6.1 Limitations

Small sample size is one of the limitations of the study. The non‐probability sampling method comes with many limitations including selection bias. This is a hospital based study conducted in dermatology clinic, so is difficult to evaluate if the population is well represented. The children who has access to hospital facilities were only included in the study. It is also possible that children with better socioeconomic status, parental occupation and education may have been enroled. In addition, this is a cross sectional study which is inferior to cohort study to investigate the associations. The larger community based studies should be conducted for further evaluation.

## CONFLICT OF INTEREST STATEMENT

The authors have no conflict of interest to declare.

## AUTHOR CONTRIBUTIONS

**Raksha Pathak**: Conceptualization (Equal); Data curation (Lead); Formal analysis (Lead); Methodology (Equal); Resources (Equal); Writing – original draft (Lead). **Sameer Shrestha**: Data curation (Equal); Formal analysis (Equal). **Prakash Poudel**: Formal analysis (Supporting); Writing – review & editing (Equal). **Suchana Marahatta**: Formal analysis (Equal); Writing – review & editing (Equal). **Dhan Keshar Khadka**: Conceptualization (Lead); Methodology (Equal); Resources (Equal); Supervision (Lead); Validation (Lead); Writing – review & editing (Equal).

## ETHICS STATEMENT

The written informed consent was taken from each participants. The ethical approval was taken from Institutional Review Commitee (IRC) BP Koirala Institute of Health Sciences. Code No: IRC/1610/019.

## Data Availability

Data openly available in a public repository that issues datasets with DOIs.
